# Leptin Levels Are Higher in Whole Compared to Skim Human Milk, Supporting a Cellular Contribution

**DOI:** 10.3390/nu8110711

**Published:** 2016-11-08

**Authors:** Sambavi Kugananthan, Ching Tat Lai, Zoya Gridneva, Peter J. Mark, Donna T. Geddes, Foteini Kakulas

**Affiliations:** 1 School of Anatomy, Physiology and Human Biology, The University of Western Australia, Perth 6009, Australia; 21141062@student.uwa.edu.au (S.K.); peter.mark@uwa.edu.au (P.J.M.); 2 School of Chemistry and Biochemistry, The University of Western Australia, Perth 6009, Australia; ching-tat.lai@uwa.edu.au (C.T.L.); zgridneva@gmail.com (Z.G.); foteini.kakulas@bigpond.com (F.K.)

**Keywords:** leptin, human milk, whole human milk, skim human milk, appetite, obesity

## Abstract

Human milk (HM) contains a plethora of metabolic hormones, including leptin, which is thought to participate in the regulation of the appetite of the developing infant. Leptin in HM is derived from a combination of de novo mammary synthesis and transfer from the maternal serum. Moreover, leptin is partially lipophilic and is also present in HM cells. However, leptin has predominately been measured in skim HM, which contains neither fat nor cells. We optimised an enzyme-linked immunosorbent assay for leptin measurement in both whole and skim HM and compared leptin levels between both HM preparations collected from 61 lactating mothers. Whole HM leptin ranged from 0.2 to 1.47 ng/mL, whilst skim HM leptin ranged from 0.19 to 0.9 ng/mL. Whole HM contained, on average, 0.24 ± 0.01 ng/mL more leptin than skim HM (*p* < 0.0001, *n* = 287). No association was found between whole HM leptin and fat content (*p* = 0.17, *n* = 287), supporting a cellular contribution to HM leptin. No difference was found between pre- and post-feed samples (whole HM: *p* = 0.29, skim HM: *p* = 0.89). These findings highlight the importance of optimising HM leptin measurement and assaying it in whole HM to accurately examine the amount of leptin received by the infant during breastfeeding.

## 1. Introduction

Human milk (HM) is a heterogeneous fluid composed of a combination of macro- and micro-nutrients, cells, and a plethora of biomolecules that provide the necessary elements to sustain infant growth, protection and development [1,2,3]. The developmental effects of breastfeeding extend to the programming of various organs and systems of the newborn, including that of appetite regulation [4]. This early developmental programming results in a reduction in obesity and other metabolic diseases not only in the short-term, but also in adulthood [5,6,7,8,9]. The complex system of breastfeeding-mediated appetite regulation is attributable to various factors associated with the practice of breastfeeding, such as feeding on demand, but also potentially to a host of appetite regulatory molecules present in HM [4]. These include whey and casein proteins, HM oligosaccharides, and recently discovered in HM appetite regulatory hormones, including the well-documented adipokine leptin, adiponectin, and many others [10,11,12,13,14].

Amongst these appetite molecules, leptin is the most widely studied, being primarily known for promoting satiety and energy expenditure in adults through binding to the full length leptin receptor (ObRb) expressed on the arcuate nucleus of the hypothalamus [15]. In addition, leptin stimulates cell proliferation, regulates blood pressure, and is also involved in the T-cell immune response, thus displaying pleiotropic roles [16,17]. White adipose tissue is one of the main sources of serum leptin, secreting leptin proportionally to the number of white adipocytes present in the body [18]. Further, gastric chief cells, the placenta, and the mammary epithelium also synthesise and secrete leptin in adults [19,20,21,22].

In infants, HM is believed to be a major source of leptin early in life, with the endogenous leptin-synthesising mechanisms being still immature [23]. Leptin in HM has been hypothesised to be involved both in the short-term control of appetite and in developmental programming of appetite and energy-signalling pathways, promoting efficient energy control and storage throughout life [9,24]. Leptin administered during the first 14 days of life has been shown to act as a neurotrophic agent, promoting neural growth from the arcuate nucleus of the hypothalamus to additional appetite control centres located in the central nervous system [25]. HM leptin may provide short-term appetite control in the infant also by up-regulating circulating melanocortins, potent anorexigenic agents that promote satiety [26]. Leptin in HM is sourced both endogenously from the mammary gland and from the maternal serum, following secretion from white adipocytes and gastric chief cells into the bloodstream [9]. In the lactating mammary gland, serum-derived leptin combines with locally-synthesised leptin by the mammary epithelium to yield the total leptin content of HM [22].

Leptin in HM has been predominately measured in skim HM, which does not contain the cellular and fat components of HM [26,27,28]. Considering that the leptin peptide is capable of lipophilic interactions [29,30], it is plausible it may associate with the fat globule in whole HM. Moreover, HM cells, which are predominantly of epithelial origin in mature HM of healthy mother/infant dyads [1,31], are also thought to contribute to the leptin concentration of whole HM [22]. Few previous studies have measured leptin in whole HM using a radioimmuno-assay (RIA) [21,32]. However, RIA is not considered appropriate for measuring leptin in a lipid-rich medium such as whole HM due to interference of triglycerides with the binding of radioactive-labelled antigens to antibodies, which compromises the sensitivity of the assay [33,34]. Given the lack of an optimised assay to detect leptin in whole HM and the absence of reliable comparisons of leptin levels between whole and skim HM, we developed an enzyme-linked immunosorbent assay (ELISA) as a more appropriate means of measuring leptin in HM, with two antibodies assisting in immobilising the leptin antigen, and compared the leptin concentration between pre- and post-feed samples, as well as whole and skim HM. 

## 2. Materials and Methods

### 2.1. Study Participants

All procedures involving the recruitment of lactating mothers and HM sample collection and analyses were approved by, and conducted in accordance with, the guidelines of the Human Research Ethics Committee of The University of Western Australia (ethics approval number RA/4/1/4253). All mothers provided informed written consent in the form of a secure online questionnaire that was administered and securely stored by The University of Western Australia. A single sample of whole HM expressed by a mother in her first month of lactation was used for optimisation of the leptin assay. Following assay optimisation, 61 lactating mothers (38 Caucasian, 23 non-Caucasian) with a mean maternal age of 33.6 ± 4.39 years, of full-term healthy infants were recruited to assess leptin differences between whole and skim HM (Table 1). HM samples (~5 mL) were obtained at approximately 1100 h aseptically, as previously described by Hassiotou et al. [35], from each breast before and after the infant fed from a single breast session either by using a Medela Symphony (Medela AG, Baar, Zug, Switzerland) breast pump or by hand expression. Samples were stored at −20 °C prior to analysis. Samples were collected at the second, fifth, ninth, and 12th months of lactation (Table 1).

### 2.2. Measurement of Leptin in Whole and Skim Human Milk by an Enzyme-Linked Immunosorbent Assay (ELISA)

Whole HM samples were thawed at room temperature, vortexed for 10 s and aliquoted (2 × 750 μL) into 1.5 mL microfuge tubes (Sarstedt, Numbrecht, Germany). One whole HM aliquot was centrifuged (05PR-22 Refrigerated Centrifuge, Hitachi, Tokyo, Japan) at 1500× *g* for 10 min at 4 °C and the resultant skim HM portion was aspirated. Both skim and whole HM aliquots were sonicated on ice at 100 Hz for three cycles of 5 s pulses, with a 20 s rest interval using an ultrasonic processor VCX130 (Sonics and Material, Newton, CT, USA). Eleven dilutions ranging from 1 to 50-fold were prepared from both milk preparations using 1% bovine serum albumin (BSA; Sigma-Aldrich, Castle Hill, NSW, Australia) in phosphate-buffered saline (PBS; Gibco Life Technologies, Paisley, Scotland).

**Figure 1:**
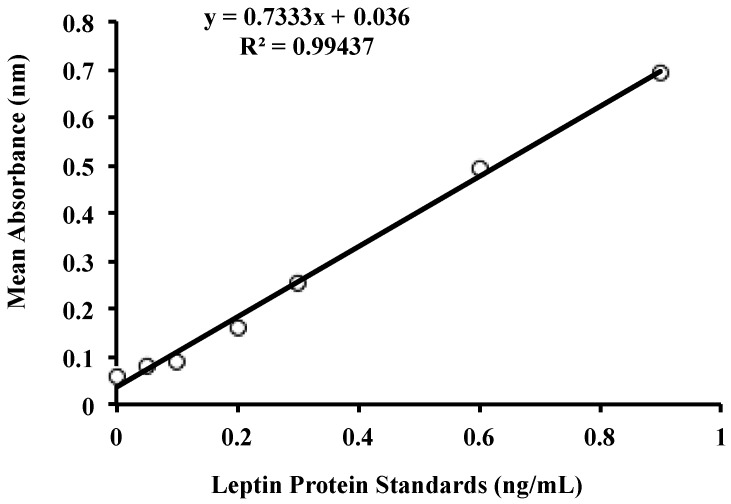
Standard curve for the leptin enzyme-linked immunoassay (ELISA) for whole and skim human milk. Standards were selected according to previous literature investigating levels of leptin in skim human milk, as well as recommendations provided by the leptin kit manufacturer [36].

**Figure 2:**
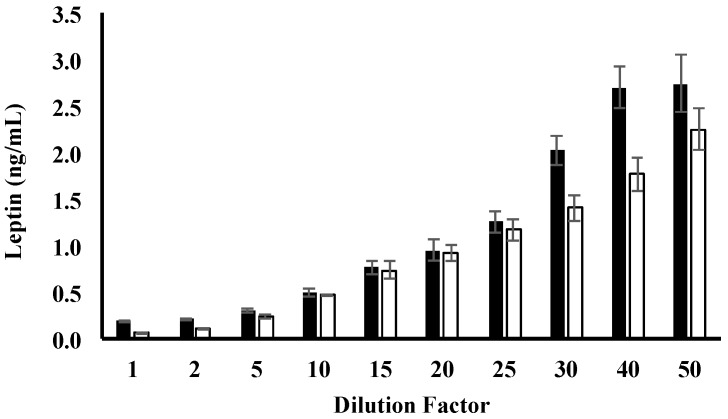
Leptin concentration for whole and skim human milk for each dilution tested. Values are mean ± SEM (*n* = 100 diluted human milk preparations). Leptin levels in whole and skim human milk are shown by black and white bars, respectively.

Leptin concentration for each dilution was measured using the Human Leptin ELISA DuoSet (R&D Systems, Minneapolis, MN, USA). Capture antibody (4 ng/mL, diluted with PBS, pH 7.4) was pipetted (100 µL per well) to coat the bottom of the wells of flat bottom 96-well microtiter plates (Flow Laboratories, McLean, VA, USA). Plates were sealed and incubated overnight at room temperature. Wells were washed three times with PBS/Tween wash buffer (0.05% Tween 20; (Bio-Rad Laboratories, Gladesville, NSW, Australia) in PBS, pH 7.4), dispensed at 400 μL per well, using a plate washer (Immunowash 1575, Bio-Rad Laboratories, Hercules, CA, USA). Washed plates were inverted and blotted against absorbent paper to ensure no remaining solution was present inside the wells. Blocking buffer (1% *w/v* BSA in PBS, pH 7.4) was added (300 μL per well) to block non-specific binding sites. Plates were sealed and incubated for one hour at room temperature. Blocking buffer was washed according to the wash procedure described earlier. Diluted samples and standards (0–0.9 ng/mL) were added (100 μL per well) in duplicates and plates were sealed and incubated for 2 h at room temperature. Unbound components from samples and standards were washed, and biotinylated detection antibody (4 ng/mL, diluted in 1% *w/v* BSA in PBS, pH 7.4) was added (100 μL per well). Plates were sealed and incubated for 2 h at room temperature. Unbound detection antibody was washed, and streptavidin-horseradish peroxidase (HRP; R&D Systems, Minneapolis, MN, USA) (50 ng/mL in PBS, pH 7.4) was added (100 μL per well), and plates were sealed, wrapped in aluminium foil to avoid exposure to direct light, and incubated for 20 min at room temperature. Streptavidin-HRP was washed and substrate colour reagent (1:1 mixture of 12 mL/vial hydrogen peroxide and 4 mL/vial enhanced luminol, R&D Systems, Minneapolis, MN, USA) was added (100 μL per well). Plates were sealed and wrapped in aluminium foil and were incubated for 20 min at room temperature. Sulphuric acid (1 M, R&D Systems, Minneapolis, MN, USA) stop solution was added (50 μL per well) and absorbance was read at 450 nm by a plate spectrophotometer (Enspire Multimode Plate Reader, Waltham, MA, USA). Standard curves and leptin concentrations were calculated using linear regression (Figure 1 and Figure 2).

Recovery assays to discern the optimal dilution factor for leptin detection were conducted on dilutions reporting leptin concentrations within the range of the protein standards used (Table 2). Following optimisation of the dilution factor, leptin concentration in matched whole and skim HM samples from the study population was measured. All whole and skim HM samples were prepared according to the same centrifugation and sonication protocol used in the assay optimisation. Recovery of a known amount of the leptin protein when added to samples was 97.7% ± 9.7% (*n* = 10) (Table 2), with the leptin kit reporting an intra-assay variability of <5% and an inter-assay variability of <7.2%.

### 2.3. Measurement of Fat Content in Human Milk

The total fat content of HM samples was measured using the creamatocrit method [37,38]. Samples were placed in micro-haematocrit tubes, plugged with sealant and centrifuged at 12,000× *g* for ten min in a micro-haematocrit centrifuge (Hermle Z230H Labortechnik, Wehingen, Germany). The resultant milk column was placed on the creamatocrit analyser (Creamatocrit Plus, Medela Inc., McHenry, IL, USA) and the length of the fat layer and the total milk column was measured, from which the total fat content was calculated. It has been shown that Creamatocrit measurements strongly correlate with the biochemical spectroscopic esterified fatty acid assay [38,39,40].

### 2.4. Statistical Analyses

Statistical analyses were performed using Microsoft Excel 2013 (Microsoft Corporation, Redmond, WA, USA) and R 2.9.10 (R Core Team, Vienna, Austria) [41] for Windows 10, with the additional R package “nlme” (R Core Team, Vienna, Austria) used for linear mixed effects modelling [42]. Student’s paired *t*-tests were conducted to assess leptin differences between matched skim and whole HM samples in the entire study population. Differences between whole and skim HM leptin concentrations were subsequently analysed within each month of breastfeeding, also using matched Student’s *t*-test.

Linear mixed effects modelling was used to examine any associations between HM fat content and whole HM leptin concentration. Responses were modelled with and without controlling for the volume of milk that had been removed from the breast during the collection of the HM sample. To discern the significant random effects to use for each statistical model analysing the association between HM fat content and whole HM leptin concentration, three separate models were devised; one linear model with no random effects and two linear mixed effects models with the following random effects: the effect of general inter-individual variation present in the study population, and the effect of the stage of lactation in addition to inter-individual variation. Analysis of variance (ANOVA) tests were then used to compare each model with the same fixed effects, namely fat content and fat content when controlled for volume of milk removed from the breast. The final model for the association between whole HM leptin concentration and fat content accounted for inter-individual variation and the stage of lactation as random effects when volume removed was not controlled for in the model. Similarly, when volume of milk removed from the breast was controlled for in the linear mixed effects models, inter-individual variation and stage of lactation were also considered as significant random variables to include in the analysis of associations between fat content and whole HM leptin concentration.

Similarly, the association between leptin concentration in whole HM samples and the corresponding fat content was also analysed within each month of lactation. Given that the volume of HM removed during feeding was only collected for 74 samples out of the entire study population, the liner mixed effects models used for intra-month analysis did not control for volume of milk removed from the breast. As with the analysis between the association of leptin levels in whole HM samples and fat content for the entire population, for each month, three statistical models were devised; one linear model with no random effects, and two linear mixed effects models with the following random variables included: the effect of inter-individual variation present in the study population and the effect of the stage of lactation in addition to inter-individual variation. Analysis of variance (ANOVA) was then used to compare each model within each month. For each month of breastfeeding, the only significant random effect found was general inter-individual variation. *p* < 0.05 was considered statistically significant. All values presented are mean ± standard deviation (SD), unless stated otherwise. All *R^2^* values were generated from the linear regression line of best fit equations.

## 3. Results

### 3.1. Participants

The demographic characteristics of mothers and infants in the study population are shown in Table 2. All infants (*n* = 61) were born at term, healthy, and were growing appropriately for their age according to the World Health Organisation’s (WHO) growth charts for exclusively-breastfed infants [43,44]. Mean maternal body mass index (BMI) was highest at 27.1 ± 7.15 kg/m^2^ during the second month of lactation and lowest during the fifth month of lactation at 23.5 ± 4.46 kg/m^2^ (Table 1). Compared to the second month of lactation, maternal BMI decreased by 2.30 ± 1.55 kg/m^2^ over the first 12 months of breastfeeding (*p* < 0.01). 

### 3.2. Leptin Optimisation

Measurement of leptin in whole and skim HM was optimised using an ELISA-based assay. One- to 20-fold dilutions for both skim and whole HM yielded leptin concentrations within the standard range of the assay (0–0.9 ng/mL), whilst dilutions above 20-fold reported values Redmond outside of the upper protein standard used (Figure 2). Mean leptin concentrations for 15-fold (whole HM: 0.8 ± 0.07 ng/mL, skim HM: 0.75 ± 0.09 ng/mL) and 20-fold (whole HM: 0.9 ± 0.11 ng/mL, skim HM: 0.9 ± 0.09 ng/mL) dilutions were close to the highest protein standard, thus further consideration was not given to these dilution factors (Figure 2). Ten-fold dilution of whole HM yielded the best recovery rates (97.7% ± 9.7%) (Table 2). Five-fold-diluted skim HM recovered 96.3% ± 1.2% of leptin (Table 2). Therefore, subsequent samples were diluted by 10-fold and five-fold with the diluent reagent for whole and skim HM samples, respectively, given these dilution factors recovered the highest percentage of leptin protein when the assay was performed for whole and skim HM. 

### 3.3. Whole and Skim Human Milk Leptin

**Figure 3:**
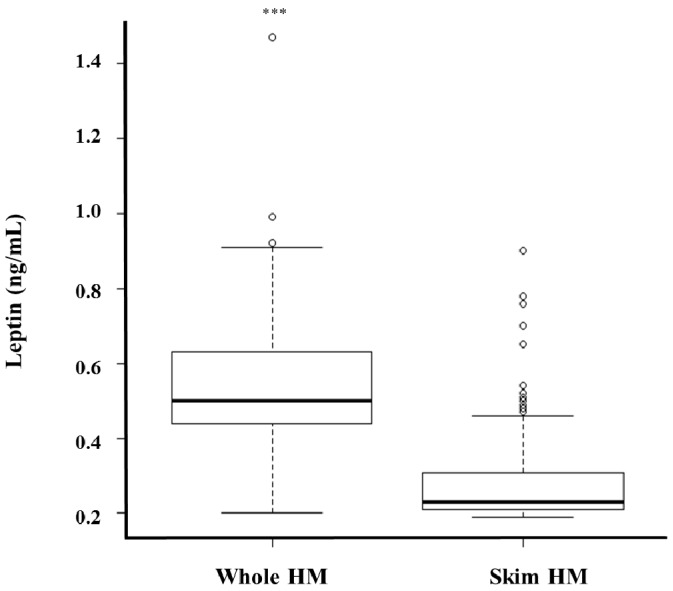
Comparison between whole and skim human milk (HM) leptin concentration (*n* = 287). *** Indicates significant difference between matched whole and skim human milk leptin values (*p* < 0.001).

Leptin levels measured using the optimised assay were compared between whole and skim HM obtained during different stages of lactation from 61 lactating mothers. Whole HM leptin levels ranged from 0.2–1.47 ng/mL, whilst a 0.19–0.9 ng/mL range was obtained for skim HM leptin (Figure 3). Whole HM leptin was 0.24 ± 0.01 ng/mL higher than skim HM leptin across all samples (*p* < 0.0001, *n* = 287) (Figure 3). Leptin levels were also higher in whole HM compared to skim HM within each month of lactation (Table 3). Matched pre-feed whole HM samples contained 0.24 ± 0.07 ng/mL more leptin than pre-feed skim preparations (*p* < 0.01, *n* = 157), with 0.25 ng/mL ± 0.05 ng/mL more leptin measured in post-feed whole HM samples compared to paired skim post-feed aliquots (*p* < 0.01, *n* = 137). 

**Figure 4:**
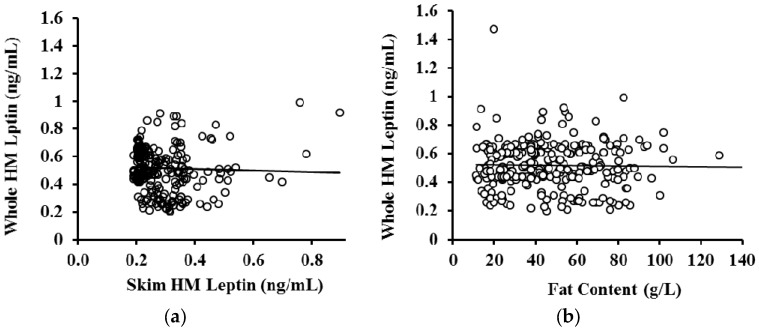
(**a**) Association between skim and whole human milk (HM) leptin (*n* = 287). No association was detected between skim and whole HM leptin levels in matched samples (*R^2^* = 0.001, *p* = 0.552); (**b**) no association was detected between fat content and leptin concentration in whole HM (*R^2^* = 0.0004, *p* = 0.17, *n* = 284). The solid black line is the line of best fit.

No association was observed between whole and skim HM leptin (*p* = 0.55, *n* = 287) (Figure 4a). HM fat content was not related to leptin concentration in whole HM when the volume of milk removed from the breast during sample collection was not accounted for (*p* = 0.52, *n* = 283) (Figure 4b) or accounted for (*p* = 0.24, *n* = 74) in the analysis. Further, no association between leptin levels in whole HM samples and fat content were found within each stage of lactation (Table 4).

**Figure 5:**
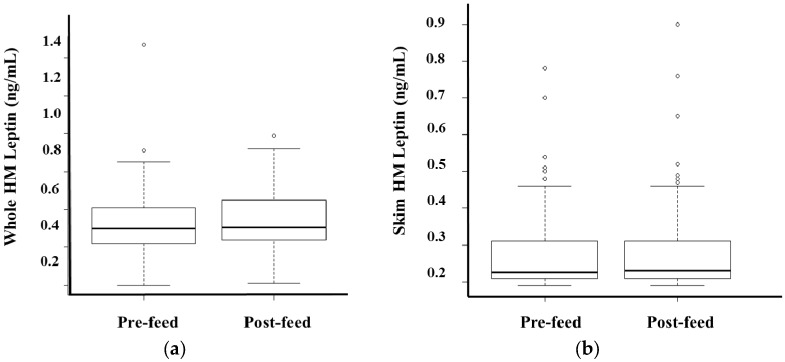
After accounting for the volume of milk consumed by the infant during the session, no differences in pre- and post-feed (**a**) whole and (**b**) skim human milk (HM) leptin values were detected in the study population.

No differences between pre- and post-feed whole (*p* = 0.29, *n* = 74) and skim (*p* = 0.89, *n* = 74) leptin levels were detected after accounting for the volume of milk consumed by the infant during the session (Figure 5). Post-feed samples contained 36.2 ± 2.82 g/L more fat compared to matched pre-feed samples (*p* < 0.01, *n* = 74).

## 4. Discussion

This study has shown that whole HM contains significantly higher levels of leptin compared to skim HM and that sampling either before or after a breastfeed does not influence this level. 

Leptin has been previously shown to be present in HM and has been hypothesised to participate in the short- and long-term regulation of appetite in the breastfed infant [9,45]. In addition, HM leptin may be involved in other functions in the breastfed infant given its known pleiotropic properties, and in mammary development [46,47]. Although many studies have previously measured leptin in HM, optimisation of the methodology has not been well documented and most studies have focused on the levels of leptin in skim HM. However, leptin has been proposed to have lipophilic properties [29] and is also synthesised by mammary epithelial cells [22], which comprise the majority of cells in mature HM from healthy mother/infant dyads [1,48,49]. It is, therefore, conceivable that leptin in HM is associated with the fat and/or cells, which are not present in its skim fraction, suggesting that previous measurements of skim HM leptin have underestimated the concentration of leptin in HM. In this study, we performed a comparison of leptin levels between whole and skim HM and optimised an ELISA assay to accurately measure it in both HM preparations.

Leptin levels were found to be, on average, two-fold higher in whole HM compared to skim HM (Figure 3, Table 3). There are limited studies that have compared whole and skim HM leptin levels and they utilized RIA based methodologies. While results of our study are in agreement with two previous studies that found higher levels of leptin in whole than skim HM, the actual values are different. Houseknecht et al. [32] reported whole HM leptin levels (10.1 ± 2.6 ng/mL, *n* = 23) that were approximately seven times higher than in skim HM (1.5 ± 0.87 ng/mL, *n* = 23) and, on average, 20 times higher than levels found in this study (Figure 3). Moreover, Smith-Kirwin et al. [21] reported 56-fold higher leptin levels in whole HM (73.2 ± 39.0 ng/mL, *n* = 8) compared to skim HM (approximately 1.3 ng/mL), and a 130-fold higher mean whole HM leptin concentration compared to the present study. For skim HM both studies observed leptin levels 1.5–2.5 times greater than those found here [21]. The higher absolute leptin levels measured in these studies may be attributable to the analysis. Both studies used the RIA technique, which is not as reliable as ELISA in measuring leptin levels in whole HM due to its known inaccuracies of immune-reactive antibodies binding to the epitopes of antigens suspended in a lipid-rich medium, such as whole HM, or of interference of iron and emulsifiers with the assay [33,34]. In addition, differences in the technique optimisation between the two previous studies are apparent, as the whole HM leptin level in one study is seven times greater than the other. Both commercially available RIA and ELISA kits are originally designed to measure leptin in serum, not in HM; therefore, optimisation is critical. On the other hand, our results are comparable to leptin levels in many other studies that measured it in skim HM using ELISA, or whole and skim HM using RIA. ELISA in skim HM detected similar leptin levels (from 0.30 ± 0.04 ng/mL at 1 month to 0.10 ± 0.02 ng/mL at 12 months, *n* = 72) in a study by Bronsky et al. [50], (0.28 ± 0.38 ng/mL, *n* = 651) in a study by Weyermann et al. [27], and (0.16 ± 0.04 ng/mL, *n* = 28) in a study by Miralles et al. [26]. RIA in skim HM also detected close leptin levels: (0.18 ± 0.15 ng/mL, *n* = 23) in a study by Schuster et al. [28] and (1.00 ± 0.80 ng/mL, *n* = 13) in a study by Schueler et al. [51]. RIA results in whole HM (1.34 ± 0.14, *n* = 24) in a study by Bielicki et al. [52] were also comparable to our whole HM leptin levels.

Whilst it has been hypothesised that leptin may be associated with fat globules present in whole HM due to its lipophilic nature [21,29] this was not borne out in this study (Figure 4b, Table 4) despite analysing pre- and post-feeding milk samples with a wide range of fat content of 11.0–128.8 g/L. The lack of an association between HM fat content and whole HM leptin levels suggests that fat may not have a strong contribution to HM leptin levels compared to the cellular fraction of HM. Lactocytes, myoepithelial cells, and stem cells present in HM have been previously shown to express the leptin gene [22]. Additionally, flow cytometric analysis of HM cells has revealed that the majority of lactocytes and stem cells contain the leptin protein [22]. Given that lactocytes are the dominant cell type in mature whole HM when both the mother and infant are healthy [1,48,49,53,54], it is likely that lactocytes contribute significantly to HM leptin levels. Indeed, the cellular fraction of HM can constitute a significant portion of milk, comparable to its skim and fat fractions [1,31]. This warrants investigation to further discern the cellular contribution to leptin levels in whole HM, and emphasises the need to assay whole HM for leptin and, potentially, for other appetite hormones, to obtain accurate measurements of the levels of these hormones in HM. Importantly, the procedures of whole HM preparation for such measurements must enable complete lysis of the milk cells for accurate results. This is also very important during sample preparation for whole HM ELISA, achieved in the present study by sonication.

Higher leptin levels in whole HM compared to the skim fraction indicate that infants ingest a larger dose of leptin than that calculated from skim HM. Whilst HM cells likely contribute to the increased leptin level in whole HM the bioavailability of this source is unknown. However, we speculate that the process of digestion would release leptin proteins from HM cells. It is also possible that these cells are absorbed through the stomach mucosa after ingestion and enter the circulation, as has been confirmed with HM leukocytes and stem cells [35,55].

The lack of an association between whole HM leptin and milk fat content may be also attributable to the biochemical properties of the leptin peptide. Although paradoxical to the notion that leptin is synthesised by white adipocytes which exhibit a lipophilic nature, leptin may also consist of hydrophilic regions, enabling it to primarily interact with aqueous fluids. The specific hydrophilic regions exhibited on the leptin peptide are hypothesised to be conserved cysteine residues tethered to disulphide bridges [29,56], which may form polar bonds with water molecules, given sulphide’s strong electronegativity properties [57]. Leptin crystallization studies could further confirm its hydrophilic properties [56], providing insight into the lack of an association between the leptin protein and the fat component of whole HM.

Upon analysis, we also found no difference between the concentration of whole HM leptin in pre- and post-feed samples, indicating the small samples taken either pre- or post-feed provide the same levels of leptin despite differences in fat content. To measure the cellular contribution of leptin in HM, it may be possible to acquire larger pre- and post-feed sample volumes and examine the number of cells in the sample. Hassiotou et al. [1] has shown that cell content increases post-feed, as does fat, when larger sample volumes were attained or the breast was well drained of milk. 

## 5. Conclusions

We describe the first standardised and optimised ELISA assay for the measurement of leptin in both skim and whole HM, demonstrating higher concentrations of leptin in whole HM preparations compared to skim HM samples. Further, we provide evidence supporting the lack of an association between the fat component of HM and its leptin content, suggesting a contribution of HM cells, which merits further investigation. Accurate analysis of whole HM leptin will assist in clarifying the biological role of this milk component for the breastfed infant, improving our understanding of early developmental programming of appetite and its implications for obesity prevention later in life.

## Figures and Tables

**Table 1:** Maternal and infant anthropometric and demographic characteristics (*n* = 61). Values are mean ± SD (range). Table includes mothers who provided samples for multiple months.

Stage of Lactation (Month)	2	5	9	12
Maternal age (years)	32.9 ± 4.21 (28–40)	33.4 ± 4.27 (24–40)	34.0 ± 4.57 (25–43)	34.1 ± 4.35 (26–44)
Maternal BMI	27.1 ± 7.15 (20.1–38.5)	23.5 ± 4.46 (18.0–35.2)	24.0 ± 5.15 (18.7–37.2)	24.8 ± 5.6 (18.2–34.6)
Parity	2.10 ± 0.75 (1–4)	2.13 ± 0.85 (1–4)	1.96 ± 0.94 (1–4)	2.05 ± 0.98 (1–4)
Infant sex (Male/Female)	12/9	16/16	17/13	12/12
Infant birth weight (kg)	3.58 ± 0.64 (2.66–4.23)	3.49 ± 0.45 (2.66–4.46)	3.49 ± 0.46 (2.82–4.46)	3.59 ± 0.46 (2.80–4.46)
Infant body length (cm)	57.6 ± 2.17 (54.2–61.3)	64.5 ± 2.09 (61.5–69.5)	70.9 ± 2.11 (68.0–74.5)	73.9 ± 2.38 (71.5–78.5)

BMI: body mass index.

**Table 2:** Recovery percentages for each dilution factor for skim and whole human milk leptin measurement.

Dilution Factor	Skim Human Milk Leptin (%)	Whole Human Milk Leptin (%)
**1**	61.5 ± 2.09	17.1 ± 2.9
**2**	179.0 ± 0.82	14.0 ± 2.7
**5**	96.3 ± 1.2	14.0 ± 1.4
**10**	71.3 ± 1.6	97.1 ± 9.1

**Table 3:** Leptin concentrations for whole and skim human milk at each month of lactation. Values are mean ± SD.

Month of Lactation	Whole Human Milk Leptin (ng/mL)	Skim Human Milk Leptin (ng/mL)	*p*-Value *
**2**	0.50 ± 0.16	0.32 ± 0.16	*p* < 0.0001
**5**	0.48 ± 0.16	0.26 ± 0.07	*p* < 0.0001
**9**	0.56 ± 0.11	0.22 ± 0.03	*p* < 0.0001
**12**	0.54 ± 0.14	0.21 ± 0.02	*p* < 0.0001

* *p*-values indicate significant differences between whole and skim human milk leptin concentrations at given time points.

**Table 4:** Association between leptin levels in whole human milk and fat content at each stage of lactation.

Month of Lactation	*N* (Samples)	*R^2^*	*p*-Value *
**2**	66	0.0013	0.782
**5**	72	0.018	0.686
**9**	83	0.069	0.577
**12**	66	0.153	0.889

* *p*-values indicate absence of associations between whole human milk leptin concentrations and fat concentrations at given time points.

## Abbreviations

The following abbreviations are used in this manuscript:

HM	Human milk
ELISA	Enzyme linked immunosorbent assay
RIA	Radioimmunoassay
PBS	Phosphate-buffered solution
BMI	Body mass index
SD	Standard deviation
SEM	Standard error of the mean
